# A Computational Approach for Predicting Role of Human MicroRNAs in MERS-CoV Genome

**DOI:** 10.1155/2014/967946

**Published:** 2014-12-23

**Authors:** Md Mahmudul Hasan, Rozina Akter, Md. Shahin Ullah, Md. Jaynul Abedin, G. M. Ahsan Ullah, Md. Zakir Hossain

**Affiliations:** ^1^Institute of Biomedical Studies, Baylor University, Waco, TX 76706, USA; ^2^BioMedNanoTech, Inc., 500 South University Avenue Suite 319, Little Rock, AR 72205, USA; ^3^Biotechnology and Genetic Engineering Discipline, Khulna University, Khulna 9208, Bangladesh; ^4^Civil Engineering Department, Bangladesh University of Engineering and Technology, Dhaka 1000, Bangladesh; ^5^Environment Science Discipline, Khulna University, Khulna 9208, Bangladesh

## Abstract

The new epidemic Middle East Respiratory Syndrome (MERS) is caused by a type of human coronavirus called MERS-CoV which has global fatality rate of about 30%. We are investigating potential antiviral therapeutics against MERS-CoV by using host microRNAs (miRNAs) which may downregulate viral gene expression to quell viral replication. We computationally predicted potential 13 cellular miRNAs from 11 potential hairpin sequences of MERS-CoV genome. Our study provided an interesting hypothesis that those miRNAs, that is, hsa-miR-628-5p, hsa-miR-6804-3p, hsa-miR-4289, hsa-miR-208a-3p, hsa-miR-510-3p, hsa-miR-18a-3p, hsa-miR-329-3p, hsa-miR-548ax, hsa-miR-3934-5p, hsa-miR-4474-5p, hsa-miR-7974, hsa-miR-6865-5p, and hsa-miR-342-3p, would be antiviral therapeutics against MERS-CoV infection.

## 1. Introduction

The new epidemic Middle East Respiratory Syndrome (MERS) has emerged since recent years. The first case of MERS was reported at September 2012 in Saudi Arabia and severity rate is increasing day by day [1]. It is caused by a type of human coronavirus called* MERS-CoV*, a new member in the lineage C of *β*-coronavirus (*β*-CoV) [2]. So far (until 15 July, 2014), 688 patients have been infected by MERS-CoV globally where 30% people already died [1, 3, 4]. Some recent studies [5, 6] indicate that bats and dromedary camels are potential host to transmit virus to human. The clinical symptoms of MERS-CoV are almost similar to Severe Acute Respiratory Syndrome Coronavirus (SARS-CoV) which emerged in 2003 [1]. Although current severity rate of* MERS-CoV* is low this scenario could be changed rapidly globally. We hope this study will play significant role in order to develop a potential antiviral therapy against MERS-CoV.

Coronaviruses are positively scene, enveloped, single stranded RNA viruses which encode 16 nonstructural proteins (nsps) including different essential and nonessential proteins [7]. However, the replication mechanism of coronavirus is still not fully clear but those proteins are thought to play vital role during viral life cycle as well as replication. Similar to other RNA viruses coronavirus replicate in the host cytoplasm. The replication process is initiated by the viral particle, after binding with their specific cellular receptors, known as S-protein mediated binding. The positive strand of RNA genome directly translated into replicase poly-proteins and further cleaved by 16 nsp [7, 8]. So the study between host and pathogen interaction always plays significant role in order to develop potential antiviral therapeutics against all coronavirus as well as MERS-CoV. Although rational vaccine design is based on the neutralization activity of highly potent antibodies since discovery of miRNAs and RNAi many of the investigators reported miRNA mediated gene silencing activity [9–11].

miRNAs are genomically encoded, small noncoding RNA molecule, generally 19–26 base pairs in length, which regulate posttranscriptional level genes expression [12–14]. It is well documented that some plants, animals, and viruses encode the miRNAs to regulate their diverse biological or physiological processes including development, apoptosis, tumorogenesis, proliferation, stress response, and fat metabolism [15, 16]. Thus 30 424 mature microRNAs have been identified from 206 species where 2578 miRNAs are encoded by human genome [17]. Virus encoded miRNAs are unique because they regulate not only their own gene expression but also their host gene expression [18].

miRNA genes are transcribed by RNA polymerase II and formed primary miRNA in nucleus. Then primary miRNAs cleaved into 60–90 base-pair-long hairpin intermediate, known as pre-miRNA, by enzymatic activity of the RNase III ribonuclease Dicer [18–20]. Pre-miRNAs are bound and exported from nucleus to cytoplasm by the action of enzyme exportin-5 and Ran (RAs-related Nuclear protein) [19]. In the cytoplasm, the pre-miRNAs are further cleaved by RNase III ribonuclease Dicer into a double stranded RNA known as duplex mature RNA [19]. Guided stand (active stand) of duplex RNA is loaded to RNA-induced silencing complex (RISC) which targets messenger RNA to degrade or repress translational activity [18]. Perfect complementarity between 3′ untranslated region (UTR) of the mRNA and the seed region of miRNA (2–7 bp) is sufficient result in cleavage but imperfect complementarity may block translation [18, 21]. Some recent study suggests that miRNA is being explored as antiviral defense against several diseases including HIV-1 [22], HSV [23], Dengue [24], Influenza [21], and hepatitis C (HCV) [25]. It has been reported that the use of miRNAs as an anti-HCV treatment demonstrated promising efficacy and safety results in an early stage trial [26]. In this study, we computationally identified some potential targets of human microRNA on Middle East Respiratory Syndrome Coronavirus (MERS-CoV) genome. Our study may help to better understand host pathogen interaction as well as to develop new antiviral therapy against MERS-CoV.

## 2. Materials and Methods

**Figure 1:**
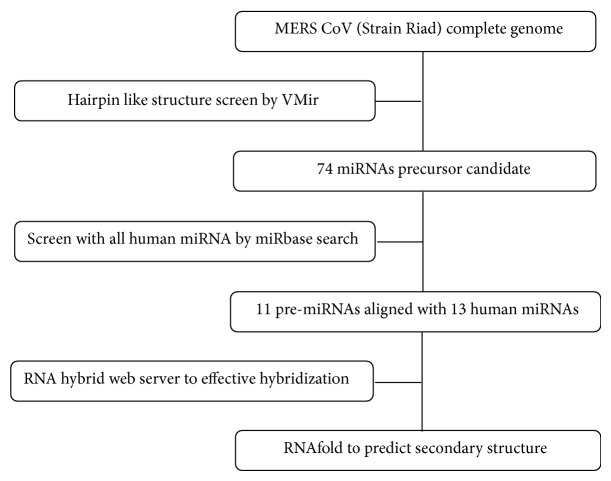
Schematic representation of human miRNA prediction on MERS-CoV viral genome [27].

The MERS-CoV miRNA prediction was carried out using the complete genome sequence of MERS-CoV (GB: KJ156952.1) obtained from the National Center for Biotechnology Information (NCBI).  Figure 1 shows a flowchart of the computational prediction process [27]. Briefly, the viral genome was scanned for hairpin-structured miRNA precursors using a VMir Analyzer program [28, 29]. VMir is an ab initio prediction program which was designed specifically to identify pre-miRNA in viral genome. The scanned hairpins were visualized in VMir viewer where 74 sequences with potential hairpin like structures were extracted as candidate miRNA precursor. Each of the sequences of the candidate miRNA precursors was searched for nucleotide similarity with all human microRNAs by using SEARCH menu of the miRBase database (http://www.mirbase.org/search.shtml) [17].

Then 11 sequences were identified as candidate miRNA precursor based on significant sequence similarity with human miRNAs. The hybridization between the viral precursor miRNAs and complementary template of the potential human miRNAs were analyzed by RNA hybrid web server (http://bibiserv2.cebitec.uni-bielefeld.de/rnahybrid/) [30]. Finally, the RNAfold web server (http://rna.tbi.univie.ac.at/cgi-bin/RNAfold.cgi) was used to predict the secondary structure of pre-miRNA [31].

## 3. Result

### 3.1. Prediction of Precursor miRNA (Pre-miRNA) Hairpins with VMir

**Figure 2:**
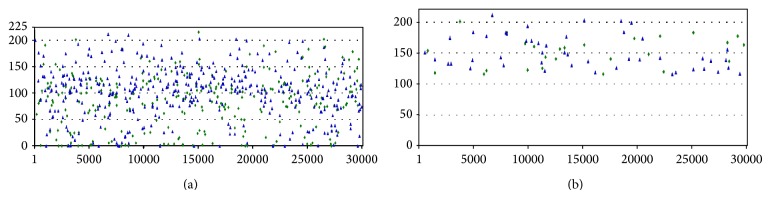
Graphical view of VMir analysis of the MERS-CoV genome. (a) All hairpins of pre-miRNAs were shown using default settings. The hairpin is plotted according to the positions of the viral genome. (b) Customized view of predicted pre-miRNA after filtering (minimum hairpin size: 60 nt, maximum hairpin size: 120 nt, minimum hairpin score: 115, and minimum window count: 25).

MERS-CoV viral genome was screened with VMir Analyzer program and the result of VMir analyzer was visualized by VMir Viewer program which shows complete output in graphical manner with sequence length and score.  Figure 2 shows the graphical representation of MERS-CoV precursor miRNAs hairpin. As default setting, 665 candidate hairpins (Figure 2(a)) have been identified. To avoid bona fide pre-miRNAs hairpin, we filtered VMir output using custom setting, that is, for cut-off value 60 nt minimum hairpin size, 220 nt maximum hairpin size, and 115 minimum hairpin score. Finally, 74 pre-miRNA hairpins (Figure 2(b)) were selected as potential hairpins for further analysis.

### 3.2. Prediction of Human miRNAs from Precursor miRNAs Hairpin

Each of the sequences of the candidate miRNA precursors was searched for nucleotide similarity with all human microRNAs by using human miRNA filter of SEARCH menu of the miRBase database (http://www.mirbase.org/search.shtml) [17, 27]. As shown in Table 1, 11 sequences were identified as candidate miRNA precursor based on significant sequence similarity with human miRNAs. Human miRNAs which show minimum 19 bp sequence similarity with candidate miRNA precursor were selected as primary target miRNAs [32]. For potential miRNA targets, near or near to perfect alignment of those miRNAs seed region (2–7) were chosen that located at the 3′ untranslated region (3′UTR) of the candidate miRNA precursor. Perfect complementarity between 3′ untranslated region (UTR) of the mRNA and the seed region of miRNA (2–7 bp) is important during gene silencing. Precursor miRNA hairpins were classified by MD (forward direction) and MR (reverse direction). Viral precursor miRNA hairpins MD5, MD17, MD110, MD157, MD186, MD244, MD366, MR175, MR201, MR268, and MR282 have shown significant identity with 13 human miRNAs. Hairpin MD110 exhibited significant sequence similarity with both hsa-miR-3934-5p and hsa-miR-4474-5p where MD186 was aligned with both hsa-miR-7974 and hsa-miR-6865-5p. Other hairpins MD5, MD17, MD157, MD244, MD366, MR175, MR201, MR268, and MR282 were aligned with hsa-miR-628-5p, hsa-miR-6804-3p, hsa-miR-4289, hsa-miR-208a-3p, hsa-miR-510-3p, hsa-miR-18a-3p, hsa-miR-329-3p, hsa-miR-548ax, and hsa-miR-342-3p, respectively.

### 3.3. Hybridization between Viral Precursor miRNAs and Human miRNAs

Effective hybridization between target human miRNA and precursor miRNA of MERS-CoV was determined by the RNAhybrid tool (http://bibiserv2.cebitec.uni-bielefeld.de/rnahybrid/) [30]. RNAhybrid is a tool for finding the minimum free energy hybridization of a long and a short RNA and widely used for microRNA target prediction. Pairing energy or minimum free energy (MFE) indicates the stability of the hybridization. For the selection of potential miRNA the pairing energy at −10 kcal/mol was utilized as cut-off score. Effective hybridizations are shown in Box 1.

### 3.4. Prediction of Secondary Structure of miRNA Precursor

**Figure 3:**
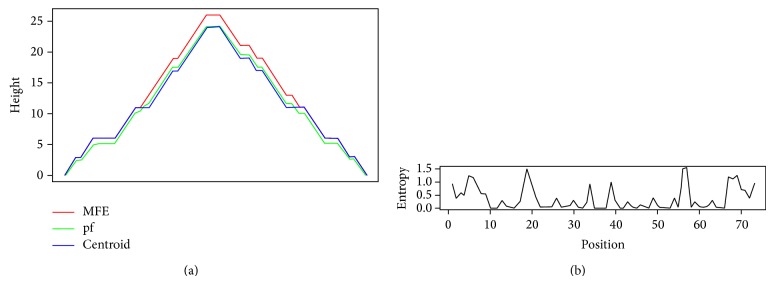
Mounting plot of predicted secondary structure of precursor miRNA hairpin. Here, hairpin MR268 was shown as an example. Red line, green line, and blue line were used to show the minimum free energy (MFE), the thermodynamic ensemble of RNA (pf), and the centroid structures, respectively.

**Figure 4:**
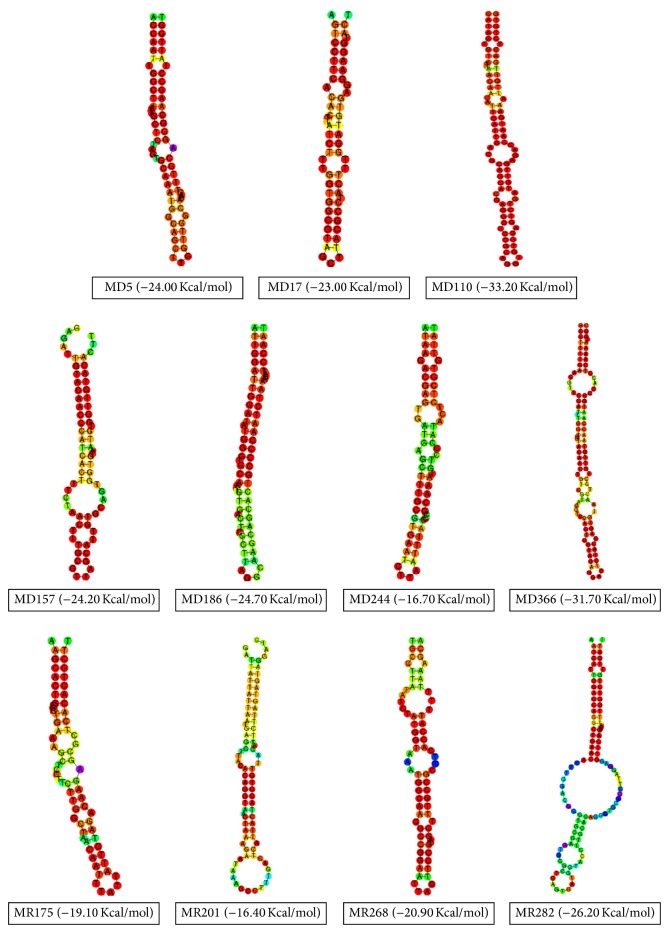
Predicated secondary structure of potential hairpins candidate of MERS-CoV. Only centroid structures were depicted.

The RNAfold web server (http://rna.tbi.univie.ac.at/cgi-bin/RNAfold.cgi) was used to predict the secondary structure of pre-miRNA (shown in Figures 3 and 4) [31]. Only default parameters were used. The RNAfold program was used to predict the most stable secondary structure of MERS-CoV Hairpin sequences. The sequence applied for prediction analysis included pre-miRNA about 200 bp upstream and about 100 bp downstream flanking sequences at each end of the precursor [27]. In all cases, folding structures with centroid were depicted.

## 4. Discussion 

MicroRNAs (miRNAs) are genomically encoded, a class of small noncoding RNAs (~22 nt), which normally function as negative regulators of target mRNA expression at the stage of posttranscriptional level [12–14, 33]. The perfect complementarity between 3′ untranslated region (UTR) of the mRNA and the seed region of miRNA (2–7 bp) is thought to be sufficient for effective cleavage but imperfect complementarity may block translation [33]. There is increasing evidence suggesting that miRNAs play critical roles in many key biological processes, such as cell growth, tissue differentiation, cell proliferation, embryonic development, cell proliferation, and apoptosis [34]. Aberrant expression of miRNAs including mutation, dysfunction, and dysregulation of miRNAs and their targets may result in various diseases, such as cancers [34, 35], cardiovascular disease [36, 37], schizophrenia [38, 39], psoriasis [40], and primary muscular disorders [41]. In the meantime, miRNAs have been great interest in utilizing miRNAs as a nonpharmaceutical approach to treat numerous diseases including HIV-1, HSV, Dengue, Influenza, and hepatitis C (HCV).

The use of miRNAs as anti-HCV treatment demonstrated promising efficacy and safety results in an early stage trial. Janssen et al. [26] reported that locking the liver-expressed microRNA-122 (miR-122) led to dose-dependent and persistent decline in HCV. By utilizing a series of bioinformatics tools, we predict 13 potential cellular miRNAs targeting the MERS-CoV virus while, except 3 miRNAs, all other predicted human miRNAs functions yet to be discovered. But based on our computational investigation, we hypothesize that those miRNAs may have significant role to know host pathogen interaction in order to develop potential therapeutics. Thus far, the 10 miRNAs, that is, hsa-miR-6804-3p, hsa-miR-4289, hsa-miR-208a-3p, hsa-miR-510-3p, hsa-miR-329-3p, hsa-miR-548ax, hsa-miR-3934-5p, hsa-miR-4474-5p, hsa-miR-7974, and hsa-miR-6865-5p, do not have any known function in human and other animals.

### 4.1. Roles of hsa-miR-628-5p, hsa-miR-18a-3p, and hsa-miR-332-3p in Humans

Those 3 miRNAs have shown significant sequence identity with MERS-CoV genome where seed region of hsa-miR-628-5p and hsa-miR-332-3p showed perfect identity with 3′ untranslated region of viral mRNA. It has been reported that hsa-miR-628-5p associated with most common brain cancer glioma and acted as protective factors where their expression decreased gradually during glioma progression [42]. Functional analysis of this miRNA indicates that they have critical roles in cell cycle and cell proliferation in glioblastoma malignant progression where has-miR-628-5p exhibited dominant regulatory activities [42].

Also, hsa-miR-18a is unregulated in basal cell carcinoma (BCC) of the skin compared with non-lesional skin [43]. Further study indicates that along with other miRNAs hsa-miR-18a target genes were predominantly involved in the regulation of cell proliferation, differentiation, and adhesion during the process of malignant transformation [44]. In addition, hsa-miR-332-3p is thought to associate with idiopathic prion disease and it has been proposed that the upregulation of hsa-miR-342-3p may be a general phenomenon in late stage prion disease and might be used as a novel marker for animal and Human Transmissible Spongiform Encephalopathies (human TSEs) [45].

## 5. Conclusion

By utilizing a series of bioinformatics tools we predict the candidate potential miRNA targeting MERS-CoV. The result suggested the miRNAs from MD5, MD17, MD110, MD157, MD186, MD244, MD366, MR175, MR201, MR268, and MR282 hairpins would be best candidate for targeting human cellular miRNAs. The utility of those 13 miRNAs, that is, hsa-miR-628-5p, hsa-miR-6804-3p, hsa-miR-4289, hsa-miR-208a-3p, hsa-miR-510-3p, hsa-miR-18a-3p, hsa-miR-329-3p, hsa-miR-548ax, hsa-miR-3934-5p, hsa-miR-4474-5p, hsa-miR-7974, hsa-miR-6865-5p, and hsa-miR-342-3p, can be utilized as antiviral therapeutics against MERS-CoV infection. However, further in vitro study should be performed in order to assess the inhibition influence on viral replication by the effect of selected human miRNAs.

## Figures and Tables

**Box 1:**
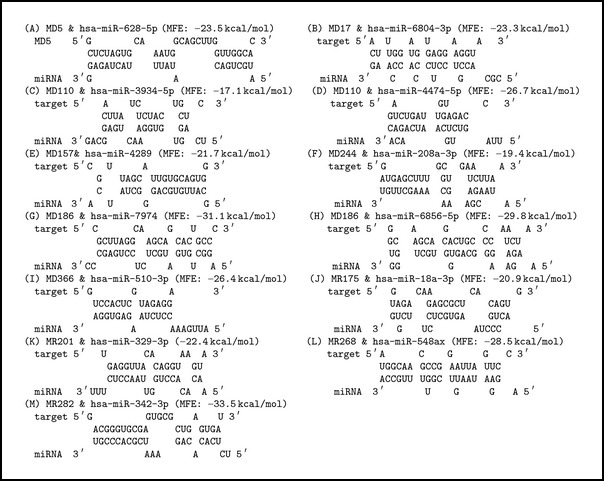
Hybridization between microRNA and viral RNA using RNA hybrid program. The program finds the energetically most favorable hybridization sites of a miRNA in a large hairpin of viral RNA.

**Table 1:** Alignments of precursor miRNAs hairpin sequences with human miRNAs.

S. number	Hairpin	Score	Alignments between human microRNA and MERS CoV			
1	MD5	151.6	UserSeq	37	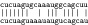	17
			hsa-miR-628-5p	1		21

2	MD17	140	UserSeq	61	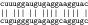	41
			hsa-miR-6804-3p	2		22

3	MD110	181.1	UserSeq	44	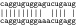	61
			hsa-miR-3934-5p	2		19
			UserSeq	71	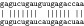	52
			hsa-miR-4474-5p	1		20

4	MD157	151.6	UserSeq	38	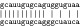	56
			hsa-miR-4289	1		19

5	MD186	151.1	UserSeq	52	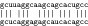	32
			hsa-miR-7974	2		22
			UserSeq	59	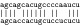	41
			hsa-miR-6856-5p	2		20

6	MD244	127.3	UserSeq	1	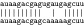	20
			hsa-miR-208a-3p	1		20

7	MD366	156.4	UserSeq	60	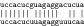	41
			hsa-miR-510-3p	2		21

8	MR175	140.9	UserSeq	5	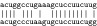	27
			hsa-miR-18a-3p	1		23

9	MR201	148.8	UserSeq	31	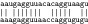	11
			hsa-miR-329-3p	2		22

10	MR268	137	UserSeq	41	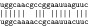	21
			hsa-miR-548ax	2		22

11	MR282	163.6	UserSeq	50	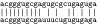	28
			hsa-miR-342-3p	1		23

## Abbreviations

MERS-CoV: Middle East Respiratory Syndrome Coronavirus
miRNA: MicroRNA
hsa-miR: Human microRNA.
